# The Youth Psychopathic Traits Inventory: Measurement Invariance and Psychometric Properties among Portuguese Youths

**DOI:** 10.3390/ijerph13090852

**Published:** 2016-08-26

**Authors:** Pedro Pechorro, Diana Ribeiro da Silva, Henrik Andershed, Daniel Rijo, Rui Abrunhosa Gonçalves

**Affiliations:** 1 School of Psychology, University of Minho, Campus de Gualtar, Braga 4710-057, Portugal; rabrunhosa@psi.uminho.pt; 2 Research Unit of the Cognitive-Behavioral Research and Intervention Center, Faculty of Psychology and Education Sciences, University of Coimbra, Rua do Colégio Novo, Coimbra 3001-802, Portugal; diana.rs@fpce.uc.pt (D.R.d.S.); drijo@fpce.uc.pt (D.R.); 3 School of Law, Psychology and Social Work, Örebro University, Örebro 70182, Sweden; henrik.andershed@oru.se

**Keywords:** adolescents, assessment, psychopathic traits, validation

## Abstract

The aim of the present study was to examine the psychometric properties of the Youth Psychopathic Traits Inventory (YPI) among a mixed-gender sample of 782 Portuguese youth (M = 15.87 years; SD = 1.72), in a school context. Confirmatory factor analysis revealed the expected three-factor first-order structure. Cross-gender measurement invariance and cross-sample measurement invariance using a forensic sample of institutionalized males were also confirmed. The Portuguese version of the YPI demonstrated generally adequate psychometric properties of internal consistency, mean inter-item correlation, convergent validity, discriminant validity, and criterion-related validity of statistically significant associations with conduct disorder symptoms, alcohol abuse, drug use, and unprotected sex. In terms of known-groups validity, males scored higher than females, and males from the school sample scored lower than institutionalized males. The use of the YPI among the Portuguese male and female youth population is psychometrically justified, and it can be a useful measure to identify adolescents with high levels of psychopathic traits.

## 1. Introduction

The construct of psychopathy is characterized by a set of affective, interpersonal, and behavioral deviant features [1,2]. Psychopathy is considered a relevant variable for forensic purposes, because it seems to be associated with the most early, severe, and stable forms of antisocial behavior [3,4,5,6]. Being a high risk condition concerning criminal recidivism, that tends to get worse and become less responsive to treatment with age, several authors argue that it is crucial to invest in early screening and intervention efforts [7,8,9,10,11,12,13]. Concordantly, the interest in child/adolescent psychopathy has vastly increased over the past decades [14]. Moreover, despite some criticisms [15,16] the more recent edition of the Diagnostic and Statistical Manual of Mental Disorders (DSM-5) [17] added the “with limited prosocial emotions” specifier for conduct disorder. This specifier includes features often identified as psychopathic traits, specifically callous-unemotional ones [17].

The Youth Psychopathic Traits Inventory (YPI) [18] is one of the available self-report screening measures to assess psychopathic traits in youth. The YPI has the advantage of assessing the psychopathic personality constellation cost-effectively using a self-report format while minimizing the possibility of deceitful answers (e.g., social desirability, response distortion) because its items were designed in an indirect and subtle way that would lead people with psychopathic traits to see them as positive or admirable [18]. Another advantage is the intended lack of items that directly tap behavioral problems or criminal conduct, since several authors argue that antisocial behavior is a product and not an inherent trait of psychopathy [1,19].

The YPI [18] was designed taking into account historical conceptualizations of psychopathy [2,20], assessing 10 core personality traits associated with the construct (grandiosity, lying, manipulation, callousness, unemotionality, impulsivity, irresponsibility, dishonest charm, remorselessness, and thrill seeking). These 10 subscales (with 5 items each) were grouped into three high-order factors, similar to the proposal of Cooke and Michie [1]: Callous-Unemotional (affective dimension: callousness, unemotionality, and remorselessness), Grandiose-Manipulative (interpersonal dimension: dishonest charm, grandiosity, lying, and manipulation), and Impulsive-Irresponsible (behavioral dimension: impulsivity, thrill-seeking, and irresponsibility) [18].

Though designed to assess psychopathic traits in youth community samples, the YPI has proven to be a good measure when it is used in forensic settings as well, maintaining its psychometric properties [21]. In the original study, Andershed and colleagues [18] used a large sample of Swedish community adolescents to assess the psychometric proprieties of the YPI. Confirmatory factor analysis supported a model with three factors: Callous-Unemotional, Grandiose-Manipulative, and Impulsive-Irresponsible [18]. Several studies reported the same three-factor structure for the YPI, either in community [22,23,24,25,26] and/or in institutionalized/forensic samples of youth [25,27,28,29,30,31]. This three-factor model has also proved to perform similarly well for boys and girls [18,22,23,25,32]. Alternatively, Pihet, Suter, Meylan, and Schmid [33] recently decided to test different models and proposed a bifactor model for the YPI, i.e., a model that simultaneously includes the total score and the three-factor scale scores.

Measurement invariance is an essential prerequisite for trustful comparisons and valid interpretations across groups, avoiding inference problems or biased/invalid conclusions [34,35]. In other words, when comparing different groups (based on age, gender, cultural background, community/clinical/forensic, etc.), it is crucial that researchers can assure that the measure assesses the same psychological construct in all groups [34,35]. Until now, only one study tested and confirmed measurement invariance of a bifactor model of the YPI (composed of the three factors’ scores and of the total score, i.e., a fourth, general factor, on which all observed variables load) across age, gender, and community vs. institutionalized samples [33]. Additionally, measurement invariance of the three-factor structure of the YPI across different ethnic groups (Dutch vs. Moroccan background) of detained male adolescents [31] was also confirmed.

The internal consistency of the YPI has been a controversial issue either with community, clinical, or forensic samples. Most studies reported a good to excellent reliability for the total score and for the Grandiose-Manipulative and Impulsive-Irresponsible factors [18,25,27,28,30,31,32,33,36]. However, there are some divergences regarding the Callous-Unemotional factor. Namely, for this factor, some authors found a poor reliability [33,36] while others found an acceptable to good reliability [23,25,26,27,31,33,37]. Reliability concerns are mainly related to Callousness, Unemotionality, Remorselessness, Thrill Seeking, Impulsiveness, and Irresponsibility subscales [30,32,33]. Particularly, Cronbach alphas have been found to be quite consistently low for the Callousness subscale across several studies [18,22,25,30,32,36].

The YPI has proven to be positively related to other measures assessing psychopathic traits, namely the Antisocial Process Screening Device (APSD-SR) [26,28,30,32,38,39], the Inventory of Callous-Unemotional Traits (ICU) [40,41,42], and even the Psychopathy Checklist: Youth Version (PCL:YV) [27,37,43,44]. Moreover, the YPI has proven to be positively linked to aggression [25,31] and, specifically, to proactive (but not reactive) aggression [26]. Externalizing psychopathology, conduct problems, risky behaviors, delinquency, criminal behavior [18,22,24,29,30,31,32], alcohol/drug abuse [23,30], and unprotected sex [30] were also positively associated with the YPI. Though the YPI has not been studied in relation to empathy measures, some studies have reported negative associations between psychopathic traits, especially callous-unemotional traits, (assessed through other measures, such as the APSD) and affective empathy [45]. Besides, the callous/lack of empathy is one of the diagnostic criteria for the “with limited prosocial emotions” specifier for conduct disorder in the DSM-5 [17]. Discriminant validity of the YPI with social anxiety has also shown mostly nonsignificant correlations [30].

Regarding gender differences, some studies reported that, generally, boys scored significantly higher than girls in all three factors of the YPI [18,22]. Moreover, comparing a male community sample of youth with a forensic sample of male young offenders, the forensic sample scored higher in the YPI and its factors [25]. However, we must state that only Pihet and colleagues’ [33] study previously tested measurement invariance of the YPI. In this study, the authors [33] found significantly higher scores in boys than in girls, as well as in institutionalized adolescents in comparison to community ones.

The YPI has been translated and psychometrically validated among an array of youth samples from different cultures and languages [18,23,30,31,36,37]. Despite this fact, the psychometric properties of the YPI have not been assessed in large, geographically diverse samples of male and female Portuguese youth while simultaneously testing for measurement invariance across gender (male vs. female) and sample type (forensic male vs. school male sample). Thus, the main goal of the present study was to validate a Portuguese version of the YPI, exploring the multidimensional structure of the psychopathy construct among male and female youth. It was predicted that: (1) the three-factor structure of the YPI would be replicated and would demonstrate measurement invariance across gender and sample type; (2) the YPI would show, in general, acceptable to good internal consistency values, as measured by the alpha and omega coefficients; (3) the YPI would show convergent validity with existing measures of psychopathic traits and aggression, and discriminant validity with measures of social anxiety and empathy; (4) the YPI scores would be positively associated with criterion-related variables such as conduct disorder symptoms, alcohol abuse, drug use, and unprotected sex; and (5) males would report more psychopathic traits than females, and males from the school sample would report fewer psychopathic traits than male young offenders.

## 2. Materials and Methods

### 2.1. Participants

The current sample was recruited from public schools of the Lisbon, Algarve, and Coimbra regions managed by the Portuguese Ministry of Education. A sample of 782 participants (*M* = 15.87 years; *SD* = 1.72 years; range = 12–20 years), subdivided into males (*n* = 371; *M* = 15.97 years; *SD* = 1.70 years; range = 12–20 years) and females (*n* = 411; *M* = 15.77 years; *SD* = 1.73 years; range = 12–20 years), agreed to voluntarily participate in the study. The participants were mostly white Europeans (89.5%). No differences were found between males and females from the school sample regarding age (*F* = 2.64 (1, 780); *p* = 0.105; η*_p_*^2^ = 0.003) nor years of education (*F* = 1.70 (1, 765); *p* = 0.193; η*_p_*^2^ = 0.193). Significant difference was found between the males from the school sample and the males from the forensic sample regarding age (*F* = 31.92 (1, 590); *p* ≤ 0.001; η*_p_*^2^ = 0.051) and years of education (*F* = 448.95 (1, 578); *p* ≤ 0.001; η*_p_*^2^ = 0.437), with the males from the forensic sample being older and having fewer years of education.

Sample type measurement invariance was examined using a previously collected forensic sample of male youth from the Portuguese juvenile detention centers managed by the Portuguese Ministry of Justice [30]. Participants in this sample included 221 male youth (*M* = 16.75 years; *SD* = 1.41 years; age range = 13–20 years). Most of them were white Europeans (54.3%), but the sample also included black Africans (20.5%), mixed race South-Americans (18.6%), and other ethnic minorities (6.8%). Most of them (87.6%) were convicted of having committed serious and/or violent crimes (e.g., robbery, assault, rape).

### 2.2. Measures

The Youth Psychopathic Traits Inventory (YPI) [18] is a 50-item self-report measure designed to assess the core personality traits of the psychopathic personality constellation in youth aged 12 years old and up. Each item is scored on an ordinal 4-point Likert scale (1 = Does not apply at all, to 4 = Applies very well). The YPI was designed in line with Cooke and Michie’s [1] three-dimensional conceptualization of the psychopathy construct, namely the Grandiose-Manipulative, Callous-Unemotional, and Impulsive-Irresponsible dimensions. Higher scores reflect an increased presence of psychopathic traits. Internal consistency based on Cronbach’s alpha has previously been reported as 0.84 for Grandiose-Manipulative, 0.74 for Callous-Unemotional, 0.78 for Impulsive-Irresponsible, and 0.88 for the YPI total [18]. The official Portuguese version of the YPI [30] was used.

The Antisocial Process Screening Device (APSD) [38] Self-Report version (APSD-SR) [46] is a multidimensional 20-item measure designed to assess psychopathic traits in adolescents modeled after the Hare Psychopathy Checklist-Revised [2]. Each item is anchored on a 3-point ordinal scale (0 = Never, 1 = Sometimes, 2 = Often). The APSD-SR has been used with preadolescents and adolescents aged 11–18 years old. Scores are calculated by reverse-scoring the appropriate reversed items and then summing the items to obtain the total score and the factors scores. This scale has three main factors: Callous-Unemotional, Narcissism, and Impulsivity. Higher scores are indicative of an increased presence of psychopathic traits. Internal consistency has previously been reported as 0.50–0.61 for Callous-Unemotional, 0.56–0.63 for Narcissism, 0.64–0.68 for Impulsivity, and 0.78–0.81 for the total APSD-SR [47]. The Portuguese version of the APSD-SR [48] was used to analyze the convergent validity with the YPI because it is presently the most used self-report measure of psychopathic traits among youths [39,48]. The internal consistencies in the current study, estimated by Cronbach’s alphas, were: APSD-SR Total = 0.77; Callous-Unemotional dimension = 0.56; Impulsivity = 0.55; and Narcissism = 0.70.

The Inventory of Callous-Unemotional Traits (ICU) [40,41] is a 24-item self-report scale designed to assess Callous-Unemotional traits in youth derived from the Callous-Unemotional (CU) subscale of the Antisocial Process Screening Device [38]. Each item is scored on a 4-point scale (ranging from 0 = Not at all true, to 3 = Definitely true). Scores are calculated by reverse-scoring the appropriate items and then summing the items to obtain the total score and the factors scores. Using confirmatory factor analysis, it was possible to identify three independent factors, namely: Callousness, Unemotional, and Uncaring, with all items also loading onto a general Callous-Unemotional factor (bifactor model). Higher scores indicate an increased presence of CU traits. Internal consistency based on Cronbach’s alpha has previously been reported as 0.70 for Callousness, 0.64 for Unemotional, 0.73 for Uncaring, and 0.77 for the ICU total [40]. The Portuguese version of the ICU [42] was used to analyze the convergent validity with the YPI because several researchers have highlighted the importance of the core affective components of psychopathy referred to as CU traits among youths [40,41]. The internal consistencies in the current study estimated by Cronbach’s alphas were: ICU total = 0.88; Callousness dimension = 0.79; Uncaring dimension = 0.84; and Unemotional dimension = 0.87.

The Reactive–Proactive Aggression Questionnaire (RPQ) [49] is a 23-item self-report measure that distinguishes between reactive and proactive aggression and is appropriate for use with youth and young adults. Each item is rated on a 3-point ordinal scale (0 = Never, 1 = Sometimes, and 2 = Often). Summed scores provide a measure of reactive or proactive aggression as well as total aggression. Confirmatory factor analysis identified two factors: Reactive Aggression and Proactive Aggression. Higher scores indicate higher levels of aggression. Internal consistency for adolescents has previously been reported as 0.86 for Proactive Aggression, 0.84 for Reactive Aggression, and 0.90 for Total Aggression [49]. The Portuguese version of the RPQ [50] was used to analyze the convergent validity with the YPI because the psychopathy construct identifies particularly aggressive and violent individuals [49,50]. Internal consistencies in the present study, estimated by Cronbach’s alphas, were: RPQ total = 0.85; Reactive dimension = 0.78; and Proactive dimension = 0.82.

The Social Anxiety Scale for Adolescents (SAS-A) [51] is a 22-item self-report scale designed to assess subjective experience of social anxiety in adolescents between the ages of 13 and 18 years. Four of the items are fillers and therefore are not taken into account in calculating the final score. Each item is rated on a 5-point ordinal scale (ranging from 0 = Not at all to 4 = All the time). Confirmatory factor analysis identified three factors: Fear of Negative Evaluation (FNE), Social Avoidance and Distress-New (SAD-New), and Social Avoidance and Distress-General (SAD-General). Higher scores indicate higher levels of social anxiety. Internal consistency based on Cronbach’s alpha has previously been reported as 0.91 for FNE, 0.83 for SAD-New, and 0.76 for SAD-General [51]. The Portuguese version of the SAS-A [52] was used to analyze the discriminant validity with the YPI because of its good psychometric properties and the fact that social anxiety generally does not overlap with the psychopathy construct [51,52]. Internal consistencies in the present study, estimated by Cronbach’s alpha, were: SAS-A Total = 0.92; FNE = 0.92; SAD-New = 0.88; and SAD-General = 0.84.

The Basic Empathy Scale (BES) [53] is a 20-item self-report measure designed to assess empathy in youth. The BES was developed as a concise and coherent scale with the aim of measuring two distinct factors: Affective Empathy and Cognitive Empathy. Each item is scored on a 5-point ordinal scale (from 1 = Strongly disagree to 5 = Strongly agree). The BES has been used with preadolescents and adolescents aged 9–18 years old. Scores are calculated by reverse-scoring the positively worded items and then summing the items to obtain the total score and the factors scores. Higher scores indicate an increased presence of empathic characteristics. The BES was validated among Portuguese youth samples, both from community [54] and forensic settings [55]. The Portuguese validation of the BES [55] was used to analyze the discriminant validity with the YPI because the extension of psychopathy to youths has highlighted the core affective components of this disorder and, given that low empathy is a core feature of the construct, it would be expected to correlate negatively with empathy [53]. The internal consistencies in the current study, estimated by Cronbach’s alphas, were: BES total = 0.92; Affective dimension = 0.89; and Cognitive dimension = 0.93.

A conduct disorder (CD) scale was also created based on the 15 items used to assess CD [56]. The 15 dichotomous items (coded 0 = No; 1 = Yes) were summated to obtain a total continuous score. Thus, higher scores indicate a higher number of positively endorsed indicators of CD. Based on the Kuder–Richardson coefficient (i.e., alpha for dichotomous items) the internal consistency of the CD scale was adequate (0.77).

A questionnaire was constructed to describe the sociodemographic characteristics of the participants. This questionnaire included variables such as participants’ age, nationality, ethnic group, and highest level of schooling completed. Some questions regarding alcohol abuse, drug use, and unprotected sex (i.e., sex without using condoms) during the last year were also included (coded as 5-point ordinal variables from 0 = Almost never/Never to 4 = Almost always/Always).

### 2.3. Procedures

Authorization to validate the YPI among Portuguese youth was obtained from the first author of the inventory [18]. The original translation of the YPI into the European Portuguese language commonly spoken in Portugal by adolescents and young adults was previously conducted [30]. During the translation and retroversion of the YPI, appropriate procedures (e.g., avoiding item bias or differential item functioning) were followed. The questionnaire was then independently back-translated into English. The original and the back-translated items were compared for nonequivalence of meaning and items were revised when any discrepancies in meaning were detected until no semantic differences were identified between the English version and the Portuguese version.

Authorization to assess youth in the school context was obtained from the General Directorate of Education of the Portuguese Ministry of Education (DGE–ME). All subjects gave their informed consent for inclusion before they participated in the study. The study was conducted in accordance with the Declaration of Helsinki, and the protocol was approved by the Ethics Committee of the DGE-ME (Code: 0338400001). Parental permission was obtained for all underage children and informed consent was obtained from participants who were 18 years of age or older. The participants—students from randomly selected public schools of the Lisbon, Algarve, and Coimbra regions—were informed about the nature of the study and asked to voluntarily participate. Not all young people agreed or were able to participate; reasons for this included refusal to participate, inability to participate due to not understanding the Portuguese language, and self-reported reading difficulties. Participants who were unwilling or unable to collaborate were excluded, so the final number of participants included in the present study was 782, with a participation rate of approximately 87%. The measures were administered in an appropriate classroom group setting using a paper–pencil method for collecting the data. The forensic sample of male youth originated from the Portuguese juvenile detention centers managed by the Portuguese Ministry of Justice, with the measures being administered by means of individual face-to-face interviews in an appropriate setting (for more details see the previous study by Pechorro and colleagues [30]).

### 2.4. Data Analysis

The data were analyzed using SPSS v23 (IBM Corp., New York, NY, USA) [57] and EQS 6.2 (Multivariate Software Inc., Encino, CA, USA). The factor structure was assessed with confirmatory factor analysis (CFA) performed in EQS 6.2 [58] with the robust estimation methods. Goodness-of-fit indices were calculated including Satorra–Bentler chi-square/degrees of freedom (S-Bχ^2^(df)), comparative fit index (CFI), incremental fit index (IFI), and root mean square error of approximation (RMSEA). A chi-square/degrees of freedom value <5 is considered acceptable, a value ≤2 is considered good, and a valued of 1 considered very good [59,60]. A CFI ≥ 0.90 and RMSEA ≤ 0.08 indicate adequate fit, whereas a CFI ≥ 0.95 and RMSEA ≤ 0.06 indicate good model fit. The incremental fit index, also known as Bollen’s IFI, is relatively insensitive to sample size where values ≥ 0.90 are considered acceptable.

The CFA was performed on the subscale scores, not on the items per se, using the same methodology as Andershed et al. [18], and only standardized loadings above 0.30 were retained. Modification indexes were considered to check for any suggestion that model modification would significantly improve the measurement model. Correlation matrixes were used together with robust methodologies to perform the CFA because they provide a more accurate estimate [61]. Measurement invariance was evaluated and the S-Bχ^2^ difference test was used to determine if the constraints significantly deteriorated the model [62]. A Microsoft Excel spreadsheet provided by Bryant and Satorra [63] was used to perform this difference test. Cronbach’s alpha (α) and omega (ω) coefficients (considered satisfactory if above 0.70), mean inter-item correlations (MIIC; considered good if within the 0.15–0.50 range), and corrected item-total correlation ranges (CITCR; considered adequate if above 0.20) were used to assess reliability [64,65]. The omega coefficient was used in the present research because it is currently considered a better estimator of reliability than alpha [66]. Pearson correlations were used to analyze associations between scale variables, and Spearman correlations were used to analyze associations between ordinal variables and scale variables [67]. Correlations were considered low if below 0.20, moderate if between 0.20 and 0.50, and high if above 0.50.

## 3. Results

Our first step in examining the psychometric properties of the YPI among the current school sample was to replicate the three-factor first-order structure proposed for this instrument by means of CFA. The following goodness-of-fit indices were obtained: male sample S-Bχ^2^/df = 3.57, IFI = 0.98, CFI = 0.98, RMSEA = 0.08 (0.07–0.10); female sample S-Bχ^2^/df = 3.48, IFI = 0.98, CFI = 0.98, RMSEA = 0.08 (0.06–0.09); and total sample S-Bχ^2^/df = 4.41, IFI = 0.99, CFI = 0.99, RMSEA = 0.07 (0.06–0.08). Based on these appropriate goodness-of-fit indices we found support for the three-factor first-order model [59,60]. We report the loadings for the three-factor first-order inter-correlated model in Table 1 for the male sample, female sample, and the combined total sample of male and female youth. All loadings were above 0.30 and, therefore, none were removed from the model. It is worth pointing out that the Callousness subscale obtained the lowest loading.

The next step was to test for measurement invariance across gender (males vs. females from the school sample) and sample type (male school sample vs. male forensic sample) using the three-factor model. We compared the configural model (no constraints included) directly with the model where factor loadings and covariances are equally constrained across groups (i.e., strong or scalar invariance) on the assumption that if strong measurement invariance holds, then weak (i.e., metric) invariance also holds. We were able to find support in terms of goodness-of-fit indices (see Table 2). The ΔS-Bχ^2^(df) values were nonsignificant in the comparison of the nested models regarding strong invariance (factor loadings and factor covariances constrained). The ΔCFI between the models was below the 0.01 cutoff. This suggests that the constraints specified do hold and leads us to assume that the models do share equivalence across gender and sample type [61].

Table 3 presents the Pearson correlations between the YPI total and its dimensions among the male sample, the female sample, and the total combined sample. As expected, mostly positive high correlations emerged.

Table 4 displays the alphas, omegas, mean inter-item correlations, and corrected item-total correlation range for the YPI among the males, females, and the combined sample. The total YPI scale and its dimensions showed good internal consistency based on alpha and omega coefficients (above the recommended cutoff value of 0.70), mean inter-item correlations (within the recommended value range of 0.15–0.50, although sometimes exceeding it), and corrected item-total correlations (above 0.20). However, some subscales, especially the ones composing the CU dimension (e.g., Callousness), showed low Cronbach’s alpha and omega coefficients, low mean inter-item correlations, or low corrected item-total correlation ranges.

Table 5 presents the correlations between the YPI and other psychometric measures and variables for the male sample, the female sample, and the total combined sample. The convergent validity of the YPI total and its dimensions with the APSD-SR, ICU, and RPQ revealed mostly moderate and high statistically significant positive correlations. The discriminant validity with the SAS-A and BES in large part revealed the expected negative or nonsignificant correlations. Table 5 also presents the correlations with CD symptoms, alcohol abuse, drug abuse, and unprotected sex. As can be seen in the table, the YPI and its dimensions showed positive statistically significant correlations with all of these behaviors that were low to moderate in terms of magnitude.

In terms of known-groups validity, a comparison of the male and female participants from the school sample revealed that the males scored significantly higher than the females, and that males from the school sample scored significantly lower than the males from the forensic sample on the YPI and its dimensions (see Table 6).

## 4. Discussion

The aim of the present study was to assess the psychometric properties of the YPI among Portuguese male and female youth from a community sample, while also testing for measurement invariance with a previously collected forensic sample [30]. The results obtained in this study using confirmatory factor analysis showed, like previous research [18,25], that the three-factor first-order model achieved an adequate fit across the three samples, namely, male, female, and total sample. The results were quite similar to the ones obtained by Pechorro and colleagues [30] in a study analyzing the YPI and the YPI-S using a forensic sample of Portuguese male young offenders, although this previously conducted study obtained slightly better results in terms of the fit indexes than the present community sample study.

Through structural equation modeling [61], the YPI showed strong measurement invariance across gender and sample type (school vs. forensic), indicating that observed scores are related to the latent scores. This suggests that the models do share some equivalence across these groups, which in turn allows for unbiased group mean comparisons [34,35,62]. This is consistent with Pihet and colleagues’ [33] study, which also revealed that the YPI is invariant across gender and sample type (community vs. institutionalized). It is important to mention that this is the first study testing for the measuring invariance of the YPI in Portuguese youths.

As expected based on previous studies [18,26,33], the associations between the YPI total and its dimensions among the male, female, and total samples showed positive high or moderate (just between the Callous-Unemotional dimension and the Impulsive-Irresponsible dimension across the three samples) statistically significant associations. These results were somewhat better than the ones previously obtained among Portuguese youths [30] that found moderate correlations (i.e., below 0.50) between the Callous-Unemotional dimension and the Impulsive-Irresponsible dimension, and between the Impulsive-Irresponsible dimension and the Grandiose-Manipulative dimension.

Measures of internal consistency for the YPI across the three samples suggested good reliability of the YPI, its dimensions, and the majority of its subscales [68], which is in line with previous studies [18,25,27,28,30,31,32,33,36]. However, like some previous research [32,36], the Callous-Unemotional dimension among the male sample showed a somewhat low Cronbach’s alpha, i.e., lower than other reported values [23,25,26,27,31,33,37]. The Callousness and the Unemotionality subscales of the Callous-Unemotional dimension and the Irresponsibility subscale of the Impulsive-Irresponsible dimension always obtained low values in terms of Cronbach’s alpha and omega coefficients across all samples. It is worth mentioning that this is the first study on the YPI, that we are aware of, using the omega coefficient, which by some is considered a better estimator of reliability than Cronbach’s alpha [66].

These results are not surprising since previous research has pointed out the same concerns within these subscales [18,22,25,30,32,33,36,44]. These results put into question the reliability of these subscales since only a small portion of the variance is attributable to them. Particularly, the Callousness subscale, as in other studies [23,28,37], reached unacceptable internal consistency values in the female and male sample. This subscale also presented low values in terms of mean inter-item/subscale correlation, and corrected item/subscale–total correlation range, suggesting a lack of homogeneity among its items. Given that the low values of the Callousness and the Unemotionality subscales of the Callous-Unemotional dimension and the Irresponsibility subscale of the Impulsive-Irresponsible dimension are not exclusive to the present study, it is possible that they are linked to particular concerns of the YPI rather than to translations or sample issues. Thus, it is conceivable that some items of these subscales need to be revised, in particular the items that are reversed, because they generally show quite low inter-item correlations. In line with this, it is interesting to notice that the Callousness subscale (the one that has presented more problems across studies), composed of five items, has all three reversed items of the YPI, which per se encompasses some limitations, including reliability issues [69,70,71].

It is noteworthy that the difficulties in assessing the affective dimension of psychopathy are not exclusive of the YPI [21], suggesting that these traits might be particularly difficult to capture. Adults and youths with high psychopathic traits tend to have a profound lack of self-insight. For example, even if they have a pronounced lack of empathy, they might affirmatively respond to questions such as “Are you a warm-hearted person?” because they generally do not see themselves as cold-hearted. This lack of self-insight poses a clear obstacle to getting valid responses to self-report items concerning some of the core psychopathic traits [18]. Despite that, the inclusion of Callous-Unemotional traits in the DSM-5 [17] as a specifier for conduct disorder, makes it a priority to: (1) improve the internal consistency of screening measures of those traits; (2) further ascertain its validity despite low internal consistency; and/or (3) create new accurate items to capture Callous-Unemotional traits [33].

The convergent validity of the YPI and its dimensions across the three samples with the APSD-SR, ICU, and RPQ revealed mostly positive moderate to high statistically significant correlations, consistent with what was expected based on previous research [26,28,30,32,39]. The associations between the BES and the affective dimension of the YPI revealed the expectable negative moderate correlations, once the affective dimension of psychopathy is, among others, characterized by a callous predisposition and a lack of empathy [9,17,45]. The discriminant validity with the SAS-A mostly revealed the expected negative or null nonsignificant correlations [30,55], due to being non-overlapping constructs [68,72]. These results were similar to the ones previously obtained by Pechorro and colleagues [30] among Portuguese youths, although this previous study did not use the RPQ and BES to assess convergent and discriminant validity, respectively.

The criterion-related validity of the YPI and its dimensions with conduct disorder symptoms [17] scored as a scale revealed moderate associations, in agreement with previous studies [18,22,24,29,30,31,32]. The highest associations were with the Grandiose-Manipulative dimension among males and total sample, and with Callous-Unemotional dimension among females. The correlations of the YPI and its dimensions across the three samples with alcohol abuse and cannabis use showed positive moderate and low statistically significant associations, consistent with previous research showing the associations between psychopathic traits and the use of these illicit substances among youth [23,30]. In terms of the correlations with the unprotected sex variable (i.e., sex without using condoms), positive low statistically significant associations were found mostly among the male sample and the total sample. Some of these outcomes (particularly the positive, though low, associations between unprotected sex and the total score and the Impulsive-Irresponsible factor of the YPI) has also been found in Pechorro and colleagues’ study [30]. Regarding the female sample, only the Impulsive-Irresponsible dimension showed a statistically significant association with this risky sexual behavior. It was interesting to find that the sizes of the correlation effect were not substantially different for many of the criteria, suggesting the possibility that the factors for many behaviors were not very discriminating, and that the YPI total score is just as useful because it is capturing the higher order construct of psychopathy.

The comparisons of male and female youth from the school sample revealed that males obtained significantly higher scores on the YPI and its dimensions. Furthermore, when comparing males from the school sample with males from the forensic sample, the results showed that young offenders obtained higher scores on the YPI total score and its dimensions. This is also consistent with Pihet and colleagues’ [33] study, which obtained similar results.

However, some limitations of this research must be mentioned. First, due to the cross-sectional nature of the study, some psychometric properties could not be evaluated (e.g., test–retest reliability). Thus, longitudinal research is needed in order to assess test–retest reliability of the YPI. A second limitation has to do with reliance on a single method for measuring the constructs (i.e., self-report), which could have resulted in high correlations due to method overlap. Future research should draw upon multiple methods in order to avoid this limitation. Another limitation was related with the use of parceling (using subscale scores rather than raw items) in factor analyses. Parceling is a psychometric technique used in the original YPI, and replicated in the present study, that unfortunately does not justify the assumption of unidimensionality. Finally, because our study was performed in a community sample, cross-validations using other samples of adolescents (e.g., clinical samples) are necessary to confirm that the results generalize to other populations. Though not particularly related to this study, for the reasons mentioned above, it would be of importance to revise some items of the YPI, specifically those related to the affective dimension, and mainly the reversed ones.

## 5. Conclusions

This was the first study investigating the psychometric properties of the YPI among a large, geographically diverse community sample of male and female Portuguese youth, while simultaneously testing for measurement invariance across gender and sample type, and reporting a more appropriate reliability coefficient. The results indicate that the YPI can be considered a useful instrument in assessing the psychopathy construct among adolescents using a self-report format. However, some caution is advised since the Portuguese validation of this promising instrument is still ongoing. We hope that our study may guide future research and practical use of the YPI with youth in Portugal and in Portuguese-speaking countries. Although the YPI was originally developed to be a tool to be used in research studies, rather than in real clinical or practical settings, it may be useful in real clinical settings as well. However, existing studies on the YPI have exclusively been based on samples recruited within research studies. Thus, to date, it is still not known how the YPI would function in real practical settings. Studies investigating this are therefore clearly needed.

## Figures and Tables

**Table 1:** Loadings for the confirmatory three-factor inter-correlated robust structure of the YPI.

	Factor 1 M/F/T	Factor 2 M/F/T	Factor 3 M/F/T
Grandiose-Manipulative dimension			
Dishonest charm subscale	0.89/0.86/0.88		
Grandiosity subscale	0.64/0.69/0.69		
Lying subscale	0.82/0.73/0.79		
Manipulation subscale	0.89/0.88/0.89		
Callous-Unemotional dimension			
Callousness subscale		0.37/0.61/0.49	
Unemotionality subscale		0.59/0.65/0.76	
Remorselessness subscale		0.90/0.83/0.99	
Impulsive-Irresponsible dimension			
Impulsiveness subscale			0.80/0.80/0.78
Thrill-seeking subscale			0.74/0.76/0.76
Irresponsibility subscale			0.74/0.68/0.72

Notes: YPI = Youth Psychopathic Traits Inventory; M/F/T = Male/Female/Total samples.

**Table 2:** Tests for invariance of the YPI goodness-of-fit statistics.

Model	S-Bχ^2^ (df)	ΔS-Bχ^2^ (df)	* CFI	* RMSEA (90% C.I.)
Cross-gender (male vs. female)				
No constraints (configural model)	244.23 (64)	--	0.98	0.08 (0.07–0.09)
Factor loadings and factor covariances constrained	262.53 (74)	18.01(10) *^ns^*	0.98	0.08 (0.07–0.09)
Sample type (school vs. forensic)				
No constraints (configural model)	168.40 (64)	--	0.98	0.07 (0.06–0.09)
Factor loadings and factor covariances constrained	181.12(74)	14.47(10) *^ns^*	0.98	0.07 (0.06–0.08)

Notes: S-Bχ^2^(df) = Satorra–Bentler chi-square (degrees of freedom); * CFI = robust Comparative Fit Index; * RMSEA = robust Root Mean Square Error of Approximation; C.I. = confidence interval; *ns* = nonsignificant at the 0.05 level.

**Table 3:** Pearson correlation matrix for the YPI and its dimensions.

	YPI Total	YPI G-M	YPI C-U	YPI I-I
Male/Female				
YPI total	1			
YPI G-M	0.92 ***/0.91 ***	1		
YPI C-U	0.76 ***/0.75 ***	0.58 ***/0.56 ***	1	
YPI I-I	0.84 ***/0.83 ***	0.64 ***/0.63 ***	0.49 ***/0.44 ***	1
Total sample				
YPI total	1			
YPI G-M	0.92 ***	1		
YPI C-U	0.79 ***	0.61 ***	1	
YPI I-I	0.83 ***	0.64 ***	0.48 ***	1

Notes: YPI = Youth Psychopathic Traits Inventory; YPI G-M = Grandiose-Manipulative dimension; YPI C-U = Callous-Unemotional dimension; YPI I-I = Impulsive-Irresponsible dimension. *** *p* ≤ 0.001 level.

**Table 4:** Cronbach’s alpha and omega coefficients, mean inter-item correlations, and corrected item-total correlations range for the YPI and its dimensions and subscales.

	Alpha	Omega	MII/SC	CI/STCR
Male/Female samples				
YPI total	0.89/00.88	0.92/0.92	0.43/0.42	0.21–0.78/0.30–0.75
G-M dimension	0.88/00.87	0.89/0.88	0.64/0.62	0.59–0.84/0.64–0.80
Dishonest charm subscale	0.82/00.80	0.83/0.80	0.47/0.44	0.43–0.71/0.42–0.67
Grandiosity subscale	0.75/00.76	0.75/0.76	0.38/0.39	0.40–0.59/0.45–0.57
Lying subscale	0.81/00.75	0.82/0.75	0.47/0.37	0.47–0.71/0.39–0.60
Manipulation subscale	0.86/00.84	0.87/0.86	0.56/0.51	0.61–0.71/0.49–0.76
C-U dimension	0.64/00.71	0.71/0.75	0.37/0.44	0.34–0.55/0.49–0.56
Callousness subscale	0.43/0.48	0.46/0.49	0.13/0.15	0.10–0.36/0.04–0.40
Unemotionality subscale	0.57/0.53	0.57/0.54	0.21/0.19	0.28–0.36/0.24–0.45
Remorselessness subscale	0.76/0.64	0.76/0.64	0.38/0.26	0.39–0.59/0.34–0.45
I-I dimension	0.80/0.79	0.81/0.80	0.58/0.55	0.61–0.69/0.58–0.69
Impulsiveness subscale	0.75/0.70	0.75/0.72	0.37/0.31	0.44–0.60/0.29–0.59
Thrill-seeking subscale	0.73/0.68	0.73/0.68	0.35/0.30	0.44–0.58/0.36–0.48
Irresponsibility subscale	0.66/0.62	0.67/0.63	0.28/0.25	0.34–0.51/0.28–0.47
Total sample				
YPI total	00.89	0.92	0.45	0.32–0.77
G-M dimension	00.88	0.89	0.65	0.64–0.83
Dishonest charm subscale	0.82	0.82	0.47	0.21–0.50
Grandiosity subscale	0.78	0.78	0.41	0.46–0.60
Lying subscale	0.80	0.80	0.44	0.46–0.64
Manipulation subscale	0.86	0.87	0.55	0.60–0.74
C-U dimension	0.72	0.76	0.46	0.48–0.59
Callousness subscale	0.54	0.56	0.19	0.10–0.45
Unemotionality subscale	0.58	0.58	0.22	0.31–0.39
Remorselessness subscale	0.72	0.72	0.34	0.38–0.53
I-I dimension	0.80	0.80	0.56	0.60–0.68
Impulsiveness subscale	0.72	0.73	0.34	0.35–0.60
Thrill-seeking subscale	0.70	0.70	0.32	0.40–0.53
Irresponsibility subscale	0.66	0.66	0.28	0.33–0.48

Notes: YPI = Youth Psychopathic Traits Inventory; G-M = Grandiose-Manipulative dimension; C-U = Callous-Unemotional dimension; I-I = Impulsive-Irresponsible dimension; Alpha = Cronbach’s Alpha; Omega = Omega coefficient; MII/SC = mean inter-item/subscale correlation; CI/STCR = corrected item/subscale-total correlation range.

**Table 5:** Correlations of the YPI total and its dimensions with other measures and variables

	YPI Total	YPI G-M	YPI C-U	YPI I-I
Male/Female				
APSD-SR	0.64 ***/0.66 ***	0.58 ***/0.54 ***	0.49 ***/0.47 ***	0.55 ***/0.64 ***
ICU	0.46 ***/0.54 ***	0.36 ***/0.42 ***	0.53 ***/0.58 ***	0.34 ***/0.40 ***
RPQ	0.51 ***/0.53 ***	0.45 ***/0.41 ***	0.36 ***/0.37 ***	0.46 ***/0.56 ***
SAS-A	0.01 *^ns^*/−0.12 *	−0.02 *^ns^*/−0.13 **	−0.04 *^ns^*/−0.14 **	0.07 *^ns^*/−0.02 *^ns^*
BES	−0.03 *^ns^*/−0.13 **	0.04 *^ns^*/−0.10 *	−0.31 ***/−0.33 ***	−0.08 *^ns^*/0.05 *^ns^*
CD symptoms	0.48 ***/0.39 ***	0.45 ***/0.32 ***	0.42 ***/0.24 ***	0.36 ***/0.41 ***
Alcohol	0.26 ***/0.41 ***	0.20 ***/0.31 ***	0.17 **/0.21 ***	0.29 ***/0.51 ***
Cannabis	0.21 ***/0.23 ***	0.17 **/0.19 ***	0.11 */0.11 *	0.24 ***/0.27 ***
Unprotected sex	0.16 **/0.08 *^ns^*	0.15 **/0.03 *^ns^*	0.14 **/−0.02 *^ns^*	0.11 */0.17 **
Total sample				
APSD-SR	0.67 ***	0.58 ***	0.51 ***	0.61 ***
ICU	0.54 ***	0.43 ***	0.59 ***	0.39 ***
RPQ	0.54 ***	0.46 ***	0.40 ***	0.52 ***
SAS-A	−0.09 *	−0.11 **	−0.13 ***	0.01 *^ns^*
BES	−0.18 ***	−0.13 ***	−0.41 ***	0.02 *^ns^*
CD symptoms	0.45 ***	0.41 ***	0.36 ***	0.38 ***
Alcohol	0.32 ***	0.24 ***	0.18 ***	0.39 ***
Cannabis	0.24 ***	0.20 ***	0.13 ***	0.26 ***
Unprotected sex	0.12 **	0.10 **	0.06 *^ns^*	0.14 ***

Notes: YPI = Youth Psychopathic Traits Inventory; G-M = Grandiose-Manipulative dimension; C-U = Callous-Unemotional dimension; I-I = Impulsive-Irresponsible dimension; APSD-SR = Antisocial Process Screening Device—Self-Report; ICU = Inventory of Callous-Unemotional Traits; RPQ = Reactive-Proactive Aggression Questionnaire; SAS-A = Social Anxiety Scale for Adolescents; BES = Basic Empathy Scale; CD symptoms = DSM-5 Conduct Disorder symptoms scored as a scale. *** *p* ≤ 0.001 level; ** *p* ≤ 0.01 level; * *p* ≤ 0.05 level; *ns* = non-significant.

**Table 6:** Descriptive statistics and ANOVAs for the YPI and its dimensions.

	*M* (*SD*)	*M* (*SD*)	*F* (*p* Value)	Effect Size η*_p_*^2^ (Power)
Cross-gender	Male school	Female school		
YPI total	54.91 (20.04)	42.12 (19.15)	83.28 (≤0.001)	0.096 (1.00)
G-M dimension	18.46 (10.26)	12.62 (9.48)	68.46 (≤0.001)	0.081 (1.00)
C-U dimension	17.77 (5.77)	12.87 (5.90)	137.17 (≤0.001)	0.150 (1.00)
I-I dimension	18.68 (7.46)	16.63 (7.36)	15.03 (≤0.001)	0.019 (0.972)
Sample type	Male school	Male forensic		
YPI total	54.91 (20.04)	70.15 (19.56)	86.69 (≤0.001)	0.124 (1.00)
G-M dimension	18.46 (10.26)	22.22 (10.51)	19.32 (≤0.001)	0.031 (0.992)
C-U dimension	17.77 (5.77)	20.11 (5.99)	23.69 (≤0.001)	0.037 (0.998)
I-I dimension	18.68 (7.46)	27.82 (7.13)	228.29 (≤0.001)	0.271 (1.00)

Notes: YPI = Youth Psychopathic Traits Inventory; G-M = Grandiose-Manipulative dimension; C-U = Callous-Unemotional dimension; I-I = Impulsive-Irresponsible dimension; *M* = Mean; *SD* = Standard Deviation; η*_p_*^2^ = partial eta squared.

## Abbreviations

The following abbreviations are used in this manuscript:

YPI	Youth Psychopathic Traits Inventory
DSM-5	Diagnostic and Statistical Manual of Mental Disorders 5th edition
APA	American Psychiatric Association
APSD-SR	Antisocial Process Screening Device – Self Report
ICU	Inventory of Callous-Unemotional Traits
RPQ	Reactive-Proactive Aggression Questionnaire
SAS-A	Social Anxiety Scale for Adolescents
BES	Basic Empathy Scale
CD	Conduct Disorder
CFA	Confirmatory factor analysis
CFI	Comparative fit index
IFI	Incremental fit index
RMSEA	Root mean square error of approximation
